# Understanding the factors influencing consumer willingness to accept the use of insects to feed poultry, cattle, pigs and fish in Brazil

**DOI:** 10.1371/journal.pone.0224059

**Published:** 2020-04-30

**Authors:** Carla Heloisa de Faria Domingues, João Augusto Rossi Borges, Clandio Favarini Ruviaro, Diego Gomes Freire Guidolin, Juliana Rosa Mauad Carrijo

**Affiliations:** 1 Federal University of Grande Dourados, Dourados, Mato Grosso do Sul, Brazil; 2 Anhanguera University – Uniderp, Campo Grande, Mato Grosso do Sul, Brazil; University of Lincoln, UNITED KINGDOM

## Abstract

The aim of this study is to investigate the factors influencing consumer willingness to accept the use of insects to feed poultry, cattle, pigs, and fish. To reach this objective, we conducted an online survey with Brazilian consumers. 600 questionnaires were collected. We analyzed data using descriptive statistics and logistic regression models. In general, the use of insects to feed poultry, pigs and cattle was not widely accepted. A more widely acceptance was found for the use of insects to feed fish. The results of logistic regressions models show that positive attitudes are associated with a higher probability of accepting the use of insects to feed poultry, pigs, cattle, and fish. Perceived benefits were associated with a higher likelihood of accepting the use of insects to feed fish. However, perceived benefits were also associated with a lower likelihood of accepting the use of insects to feed poultry. Perceived challenges were associated with a higher likelihood of accepting the use of insects to feed poultry. However, perceived challenges were associated with a lower likelihood of accepting the use of insects to feed pigs.

## Introduction

The increase in the world’s population will cause a higher demand for animal-source food, which will require a boost in the production of proteins, because proteins are important components of animal feed [1]. A higher production of proteins, however, might contribute to the depletion of environmental resources [2]. Furthermore, protein is one of the most expensive and limiting ingredients to feed animals [3, 4]. In this scenario, the use of insects as an alternative source of proteins in animal feed could be a solution because of their high nutritional value, high protein level, low level of greenhouse gas emissions, and the little amount of water required to produce insects compared to common crops [1–6]. Despite such potential benefits, a lack of a clear legislation and the uncertainty about consumer reactions are major sources of constraints to a widespread use of insects in animal feed [7]. The introduction of insects in animal feed has also raised challenges about food safety issues, including allergies in animals and humans, chemical and microbial contaminants, and the nutritional benefits of edible insects for both humans and animals, most of which need further investigation [8].

The regulatory system on the use of insects in animal feed differs between countries. The European Union currently authorizes the use of processed animal proteins (PAP) derived from insects in aquaculture [9], but European Commission services are exploring possibilities to authorize PAP for poultry feed [8]. In the US, edible insects are considered food additives and currently only the black soldier fly (*Hermetia illucens*, HI) has been included as an ingredient in animal feed, and its use is restricted to aquaculture [9]. In Canada, the use of HI larvae is authorized for aquaculture and poultry. In China and South Korea, there are no specific regulations [9]. Brazil has also not developed a specific legislation on the use of insects in animal feed, but PAPs are allowed in diets to feed non-ruminant animals [10].

Regarding consumer reactions to the use of insects in animal feed, a major concern is that in general humans avoid unfamiliar foods (i.e., neophobia), particularly from animal origins [1, 11]. The implementation of insects as food and feed is particularly challenging in Western cultures because consumers neither consider insects as food nor consider insects appropriate for consumption [12]. Previous research conducted in Western and Eastern cultures has focused on consumer willingness to replace meat for insects [2, 5, 13–17]. Although we acknowledge the contribution of such studies, we agree with other authors that argue that insects could be introduced in consumers’ daily diet more easily by developing products that are already currently consumed [3, 12, 18] or by using insects in animal feed.

Specifically, studies on consumer preferences and barriers for the use of insects in animal feed are scarce [1, 9]. Verbeke et al [2] in research conducted in Belgium, investigated consumer acceptance of using insects in animal feed. Their results showed that the use of insects to feed fish and poultry was widely accepted. In the same study, Verbeke et al. [2] found that consumers have a critical attitude towards the use of insects to feed cattle for either milk or beef. La Barbera et al [19] in research conducted to understand the main drivers and barriers related to Western consumers’ acceptance of food with ingredients derived from insects found that the acceptance of indirect entomophagy do not indicate the acceptance of direct entomophagy due to it may portray insect-based food as “good for animals, bad for humans”.

Brazil is one of the main producers and exporters of animal-source food and the greatest feed protein supplier in the world. Most of the protein used to feed animals comes from common sources (e.g., soybean) [20]. Therefore, if the world wants to succeed in implementing the use of insects in animal feed, Brazil plays an important role in it. However, little is known about how consumers in Brazil will react to the introduction of insects in animal feed.

Considering the foregoing, the aim of this study is to investigate the factors influencing consumer willingness to accept the use of insects to feed poultry, cattle, pigs, and fish. Such factors include consumer attitudes towards using insects in animal feed, perceived benefits, perceived risks, perceived challenges about the use of insects in animal feed, and socio-demographic characteristics. We believe that such a study can provide insights to policy makers and private companies that can be used to develop strategies to increase the acceptance of the use of insects to feed poultry, cattle, pigs, and fish.

## Material and methods

### Survey and sampling

We developed four similar questionnaires. Each questionnaire focuses on a specific animal (i.e., poultry, cattle, pigs, and fish). The questionnaires consisted of four groups of questions adapted from Verbeke et al. [2]. In the first group, we measured socio-demographic characteristics, previous contact with the specific animal, and willingness to accept the use of insects in animal feed (see S1 Table for details). In the second group, we measured general attitudes towards rearing insects instead of crops to use in animal feed, and attitudes towards using insects to feed specific animals (poultry, cattle, pigs, and fish) (see S2 Table for details). In the third group, we used statements to measure perceptions related to five possible benefits and seven possible risks associated with the use of insects in animal feed (see S3 Table for details). In the fourth group, we used statements to measure challenges facing the introduction of insects in animal feed (see S4 Table for details). The survey was extensively pre-tested and refined prior to application. All questions were translated into Portuguese. This project was approved by the Research Ethics Board of the Federal University of Grande Dourados/Faculty of Management, Accounting and Economics.

To collect data, we conducted an anonymous online survey. The survey was distributed in all regions of Brazil. The sampling and the application of the survey were performed using a specialized market research company, which has its own panel of respondents. Registration in the panel is voluntary and recruitment of participants occurs by advertisements in social media by people who find the company’s website themselves or by people who are referenced to by other users. In our research, respondents were randomly selected in the panel and received an invitation to participate in the survey. Upon acceptance, they received incentives (e.g., points in loyalty programs or bonuses to be used in mobile phone services). To ensure the necessary level of rigor, we monitored and commented on each step of the sampling and survey implementation. 600 questionnaires were collected, 150 for each of the four animals. The data collection took place in March 2018.

### Statistical analysis

Statistical analysis was conducted in three steps. In the first step, we used descriptive statistics to characterize the respondents and the main measures. In the second step, we used factor analysis to reduce the number of items that represent consumer attitudes, perceived benefits, perceived risks, and perceived challenges about the use of insects in animal feed. Principal component analysis was used as the extraction method. The criterion to define the number of factors was an eigenvalue greater than one [21]. Items were included in a factor when they presented factor loadings greater than 0.5. Factors scores were generated for subsequent analysis [21]. We used factors scores to represent attitudes, perceived benefits, perceived risks, and perceived challenges because items receive different weights in the composite and, as the factor scores were rotated, little variance overlap among factors scores.

In the third step, we ran four logistic regression models. The dependent variable was consumer willingness to accept the use of insects in animal feed. We tested the impacts of five groups of independent variables: socio-demographic characteristics, attitudes, perceived benefits, perceived risks, perceived challenges about the use of insects in animal feed. The significance level was p<0.05. We assessed multicollinearity by running multiple regressions, each with a different item as the dependent variable and the rest of the items as independent variables, and then verifying the tolerance and variance inflation factor (VIF) [22]. We found a high multicollinearity between the items that measured general attitudes and the variables that measured attitudes towards the use of insects to feed specific animals. Thus, we decided to maintain in the analysis only the variables that measure attitudes towards using insects to feed specific animals.

## Results

### Descriptive statistics

Descriptive statistics are presented in Table 1. In the questionnaires, the socio-demographic characteristics of the respondents were similar, except for gender, income and type of contact with specific animals. In the poultry and fish questionnaires, most respondents were male. The samples in the poultry and cattle questionnaires had a lower income compared to samples in the pig and fish questionnaires. The type of contact with different animals was similar between poultry and fish questionnaires and between cattle and pig questionnaires. In general, the use of insects to feed poultry, pigs and cattle was not widely accepted; nearly half of the respondents favored this idea and half did not. A widely acceptance was found in the use of insects to feed fish: two thirds of the respondents favored this idea.

**Table 1:** Descriptive statistics of the socio-demographic and ‘willingness to accept’ variables used in the poultry, cattle, pig and fish questionnaires.

Variable	Poultry (%)	Cattle (%)	Pig (%)	Fish (%)
Age (years) (mean and standard deviation in brackets)	33 (1.02)	34 (1.07)	33 (1)	35 (1.05)
Gender:				
1: male	52.67	42.67	48	52
2: female	47.33	57.33	52	48
Income:				
1: more than R$ 14,970.00	2	2.67	2	2
2: R$ 4,990.00 –R$ 14,970.00	16	16	21.33	20
3: R$ 2,994.00 –R$ 4,970.00	32	29.33	28.67	34.67
4: R$ 998.00–R$ 2,994.00	30.67	32.67	26.67	24.67
5: R$ 998.00	19.33	19.33	21.33	16.67
Educational level:				
1: incomplete elementary school	4	4	4	5.33
2: complete elementary school	3.33	5.33	2	2
3: incomplete high school	8	7.33	10	6.67
4: complete high school	41.33	36	33.33	34.67
5: incomplete bachelor’s degree	21.33	22	18.67	16.67
6: complete bachelor’s degree	14.67	14.67	20	25.33
7: incomplete postgraduate studies	1.33	2	1.33	2
8: complete postgraduate studies	6	8.67	10.67	7.33
Local of residence:				
1: urban	89.33	80.67	86.67	86.67
2: rural	4.67	4.67	0.67	2.67
3: both	6	14.67	12.67	10.67
Region:				
0: South and Southeast	60.67	60	58	54.67
1: Midwest, Northeast and North	39.33	40	42	45.33
Contact with the animal supply chain:				
0: no	34.67	46.67	38	10.67
1: yes	65.33	53.33	62	89.33
Type of contact with the animal supply chain:				
1: I lived in a rural propriety that produced broilers ^a^	12.24	12.50	12.90	7.46
2: Someone in the family has or had a rural property that produces broiler ^a^	27.55	46.25	49.46	24.63
3: I visited rural properties that produced broilers ^a^ but I have never had direct contact with these animals	32.65	23.75	27.96	35.82
4: I work or worked in poultry ^a^ supply chain	7.14	8.75	1.08	3.73
5: other	20.41	8.75	8.6	28.36
Willingness to accept the use of insects in poultry ^a^ feed:				
0: no	44.67	50	44.67	24.67
1: yes	55.33	50	55.33	75.33

^a^ The words ‘poultry or broiler’ were replaced for the word ‘beef or cattle’ in the beef questionnaire, for the words ‘pigs or pork’ in the pigs questionnaire, and for the word ‘fish’ in the fish questionnaire.

### Factor analysis

The results of factor analysis showed an eigenvalue above 1.0 for the items measuring attitude, perceived benefits, perceived risks, and perceived challenges. The same pattern occurred in the analysis of data from the four questionnaires. We decided to remove one item measuring perceived risk due to its cross factor loading. The item was excluded from the analysis of data of the four questionnaires. The item was ‘The use of insect-based meal in animal feed can increase competitiveness with other agricultural activities.’

By adapting from Verbeke et al. [2], we created one factor to represent ‘Attitude’ (Att), one factor to represent ‘Perceived benefits’ (PB), one factor to represent ‘Perceived risks’ (PR), and one factor to represent ‘Perceived challenges’ (PC) about the use of insects to feed poultry, cattle, pigs, and fish.

Descriptive statistics of the statements used to measure attitudes, perceived benefits, perceived risks, and perceived challenges are presented in S5–S7 Tables, respectively. For statements measuring attitudes towards the use of insects to feed poultry, cattle, pigs, and fish (S5 Table, Att 9–12), the means were below or close to 3, which indicates that respondents have a neutral attitude. For statements measuring perceived benefits and perceived risks about the use of insects to feed poultry, cattle, pigs, and fish (S6 Table, PB1-5; PR1-7), the means were slightly above or close to 3, which indicates that individuals were neutral about the possible benefits and possible risks. For statements measuring perceived challenges about the use of insects to feed poultry, cattle, pigs, and fish (S7 Table, C1-10), the means were a little higher than 3, which indicates that individuals were neutral about it.

### Logistic regression models

We tested whether socio-demographic characteristics, attitudes, perceived benefits, perceived risks, and perceived challenges could affect consumer willingness to accept the use of insects to feed poultry, cattle, pigs, and fish. Table 2 presents the results of the four logistic regression models with the estimated logistic regression coefficients (β), their respective standard errors (SE), significance level, and Exp (β), which are the odds ratio between the probability of a person accepting or not the use of insects in animal feed.

**Table 2:** Logistic regression models of the willingness to accept the use of insects in feed for poultry, cattle, pigs and fish supply chains.

Independent variables	Willingness to accept the use of insects in poultry feed ^a^			Willingness to accept the use of insects in cattle feed ^b^			Willingness to accept the use of insects in pigs feed ^c^			Willingness to accept the use of insects in fish feed ^d^		
	Β	S.E.	Exp (β)	β	S.E.	Exp (β)	β	S.E.	Exp (β)	Β	S.E.	Exp (β)
Age	-0.036	0.027	0.963	0.020	0.024	1.021	0.029	0.023	1.029	-0.110*	0.054	0.895
Gender	-1.553	0.827	0.211	-0.078	0.633	0.924	0.399	0.538	1.490	-1.172	1.136	0.309
Region	-0.277	0.787	0.757	-1.624*	0.659	0.197	-0.327	0.534	0.720	-0.434	0.966	0.647
Income	-0.671	0.395	0.511	-0.210	0.369	0.810	0.133	0.274	1.142	-1.294*	0.646	0.274
Educational level	-0.914	0.276	0.823	0.017	0.207	1.017	0.300	0.193	1.350	0.058	0.311	1.060
Contact with the animal supply chain	-0.088	0.722	0.914	0.642	0.560	1.902	1.334*	0.564	3.799	2.550	1.409	12.816
Attitude toward using insects in animal feed	6.602*	1.556	737.2	2.781*	0.714	16.133	1.737*	0.508	5.684	2.573*	0.947	13.107
Perception of benefits associated with the use of insects in animal feed	-1.790*	0.820	0.166	0.406	0.607	1.501	0.793	0.465	2.210	2.521*	1.111	12.441
Perception of risks associated with the use of insects in animal feed	0.232	0.431	1.261	-0.628	0.432	0.533	-0.334	0.347	0.715	-1.023	0.744	0.359
Challenges facing the introduction of insects in animal feed	1.438*	0.518	4.215	0.311	0.358	1.365	-0.657*	0.328	0.518	0.234	0.630	1.264
Constant	7.985	2.907	2937	0.551	2.247	1.736	-3.810	1.986	0.022	11.582	5.306	1071
Likelihood logarithm	-29.859			-42.665			-51.810			-20.909		
Chi-square value	146.52			122.610			102.61			125.77		

* p <0.05.

^a^ Goodness-of-fit statistics of the model “Willingness to accept the use of insects in poultry feed”: Nagelkerke R^2^ = 0.69; % of correct predictions = 91.3%.

^b^ Goodness-of-fit statistics of the model “Willingness to accept the use of insects in cattle feed”: Nagelkerke R^2^ = 0.59; % of correct predictions = 89.3%.

^c^ Goodness-of-fit statistics of the model “Willingness to accept the use of insects in pigs feed”: Nagelkerke R^2^ = 0.50; % of correct predictions = 86.0%.

^d^ Goodness-of-fit statistics of the model “Willingness to accept the use of insects in fish feed”: Nagelkerke R^2^ = 0.75; % of correct predictions = 94.0%.

The socio-demographic characteristics gender and educational level did not affect consumer willingness to accept the use of insects to feed poultry, cattle, pigs, and fish. A one-year increase in age was associated with a 10% decrease in the likelihood of accepting the use of insects to feed fish. Living in the Midwest, Northeast and North of Brazil was associated with an 80% decrease in the likelihood of accepting the use of insects to feed cattle. A one-unit increase in income was associated with a 73% decrease in the likelihood of accepting the use of insects to feed fish. Consumers who indicated previous contact with pig farms were 3.8 times more likely to accept the use of insects to feed pigs than those who did not indicate a previous contact. Positive attitudes were associated with a higher probability of accepting the use of insects to feed poultry, pigs, cattle, and fish. Consumers who perceived higher benefits were more likely to accept the use of insects to feed fish than those who perceived lower benefits. However, consumers who perceived higher benefits were less likely to accept the use of insects to feed poultry than those who perceived higher benefits. Consumers who perceived fewer challenges were more likely to accept the use of insects to feed poultry than those who perceived more challenges. However, consumers who perceived more challenges were more likely to accept the use of insects to feed pigs than those who perceived fewer challenges.

## Discussion and concluding comments

This is the first study investigating consumer willingness to accept the use of insects in animal feed in Brazil. Our results contribute to the existing literature because previous studies have shown that consumer willingness to accept new food technologies, such as the use of insects in animal feed, depends on the country where the study is conducted [23–26].

We found a higher acceptance of the use of insects to feed fish compared to poultry, pigs and cattle. The use of insects to feed cattle presented the lowest acceptance rate. These results are somehow in line with Verbeke et al. [2], who also found that Belgium consumers were more willing to accept the use of insects to feed fish and poultry than to feed cattle. A possible explanation for such results is that it is easier to accept that insects could be used to feed poultry and fish since these animals have access to and might eat insects in their natural environment [2]. Such argument might explain the higher acceptance of the use of insects to feed poultry and fish compared to cattle, but not to explain the higher acceptance of the use of insects to feed pigs than to feed cattle. In the Brazilian context, a possible explanation is that beef is more consumed than pork and therefore consumers are more willing to accept the use of insects to feed pigs because they will not regularly consume it. It is interesting to note that according to Brazilian legislation, PAPs are allowed in diets to feed non-ruminant animals, and our results showed that consumers accepted more the use of insects to feed fish, poultry and pigs than to feed ruminants (i.e., cattle). Following a proper regulation, this situation indicates that, in Brazil, PAPs derived from insects might be more easily introduced in the diet of non-ruminant animals.

Interestingly, the average scores for all items measuring attitudes, perceived benefits, perceived risks, and perceived challenges are in a central position (close to 3 in a scale from 1 to 5) for the four animals analyzed. There are some possible explanations for these results. First, when a Likert-scale with a neutral response option is used people might choose the neutral point to avoid the cognitive effort required to pick a satisfactory answer, which is called satisfice [27]. Secondly, people pick the neutral point to avoid the cognitive effort needed to choose between their positive and negative feelings on an issue, which is called ambivalence [28]. Third, people might choose the neutral point because of social desirability [27]. Finally, people choose the neutral point when they have not formed attitudes and opinions about a specific issue. Although it is not possible to test the impacts of satisfice, ambivalence and social desirability in this study, we speculate that neutral scores occurred because the use of insects in animal feed is a new concept for consumers, and therefore they simply might not have an already well established attitude and do not have an opinion about the potential benefits and risks.

The results of the logistic regression models were slightly different, indicating that the factors influencing consumers’ willingness to accept the use of insects in animal feed depend on animal (i.e., poultry, pigs, cattle, and fish) that will feed on insects. In general, socio-demographic characteristics seem not consistent to explain consumer willingness to accept the use of insects in animal feed because no socio-demographic variable that we tested had a significant impact in all four logistic models. Instead, age and income were significant in explaining consumer willingness to accept the use of insects to feed fish, with an increase in age and an increase in income being associated with a lower likelihood of accepting the use of insects to feed fish. These results might be explained because older individuals with a lower income are more neophobic; they are more prudent and seek safer and known foods [2]. Consumers who live in the Midwest, Northeast and North regions of Brazil were less willing to accept the use of insects to feed cattle compared to those who live in the South and Southeast regions. This result might be explained by differences in cultures among these regions. Indeed, previous studies have shown that consumer rejection to new food technologies depends on food taboos, which are usually acquired by sociocultural factors [5, 15, 29]. For instance, exposure and social learning affect people’s choices about what is appropriate to eat and which foods they are supposed to like [5]. As the South and Southeast regions of Brazil are more developed than the Midwest, Northeast and North regions, it is reasonable to assume that consumers who live in the South and Southeast have more information about new food technologies as well as more contact to different types of food, which might keep them open-minded to the use of insects in animal feed.

The results of the logistic regression models showed that consumer attitude towards the use of insects in animal feed consistently explain consumer willingness to accept the use of insects in animal feed. These results are in line with previous literature that found that individuals holding more positive attitudes were more willing to accept new food technologies [1, 2, 5, 9, 26]. This result is important because personal attitudes related to the use of insects in animal feed might outweigh the adverse impacts of perceived risks and perceived challenges related to it [2].

The results of the logistic regression models also showed that perceived benefits affect consumer willingness to accept the use of insects to feed poultry and fish. Surprisingly, consumers who perceived more benefits were less willing to accept the use of insects to feed poultry. This result is hard to explain. A possible explanation is that the use of insects as an alternative source of protein is new and unfamiliar, therefore consumers might not have a clear picture of the possible benefits presented in the questionnaire. Indeed, according to Napier et al. [30], most consumers are unable to decide and are hesitant to accept new food technologies when it is associated with unclear benefits. In contrast, our results show that consumers who perceived more benefits are more willing to accept the use of insects to feed fish. This result is in line with that found in the literature showing that the more consumers perceive the benefits of a new product, the higher the willingness to accept it [1, 2, 5, 18, 26].

In logistic regression models we also found that perceived challenges affect consumer willingness to accept the use of insects to feed poultry and pigs. Once again, results for poultry are difficult to interpret because consumers who perceived more challenges were more willing to accept the use of insects to feed poultry. A possible explanation is that individuals who are presented to unfamiliar food technologies might not understand them, causing some resistance and challenges [26]. However, we found that consumers who perceived fewer challenges were more willing to accept the use of insects to feed pigs.

From a private and public policy perspective, our results provide insights that can be used to design strategies to increase the acceptance of the use of insects in animal feed. The strong and consistent impact of attitudes on consumer willingness to accept highlights the importance of design strategies to disseminate the benefits of using insects to feed animals. For instance, we believe that important benefits disseminated by information campaigns are, for instance, ‘the use of insects in animal feed decrease environmental impacts of food production’ and ‘the use of insects in animal feed increases animal productivity.’ In addition, the academia and industries should collaborate closely to develop further research and technology related to the use of insects in animal feed and the population should be engaged in this process, which might increase the willingness to accept this technology.

A potential limitation of our study is that online representativeness is not similar as the representativeness of the whole Brazilian population. Hence, our sample cannot be considered fully representative of the Brazilian population. Therefore, our findings must be considered primarily as exploratory. Another limitation was that consumers might not have enough information to evaluate the use of insects in animal feed. Therefore, it is possible that respondents faced difficulty in predicting their willingness to accept, their attitudes, perceived benefits, perceived risks, and perceived challenges about the use of insects in animal feed.

Because we found contradictory results about the impacts of perceived benefits and perceived challenges on consumer willingness to accept the use of insects in animal feed, we recommend that future studies further explore these issues. We believe that a clearer picture should emerge when information about the use of insects in animal feed becomes widely available for consumers in Brazil. Future studies might also benefit from the use of recent standardized questionnaires developed to measure consumer attitudes towards the use of insects in animal feed [19].

## Supporting information

S1 Table Sociodemographic and “willingness to accept” variables, questions and scales used in the questionnaires. (DOCX)

S2 Table Questions and scales used to measure attitude variables. (DOCX)

S3 Table Questions and scales used to measure the perceived benefits and perceived risks variables. (DOCX)

S4 Table Questions and scales used to measure perceived challenges variables. (DOCX)

S5 Table Descriptive statistics of attitude items used in the poultry, cattle, pig and fish questionnaires. (DOCX)

S6 Table Descriptive statistics of perception items used in the poultry, cattle, pig and fish questionnaires. (DOCX)

S7 Table Descriptive statistics of challenges items used in the poultry, cattle, pig and fish questionnaires. (DOCX)
