# Supplementary material for: Understanding the factors influencing consumer willingness to accept the use of insects to feed poultry, cattle, pigs and fish in Brazil
Source: PLoS One. 2020 Apr 30;15(4):e0224059. doi: 10.1371/journal.pone.0224059 (PMC7192463; doi:10.1371/journal.pone.0224059)
Supplement: S1 Table — (DOCX) [file pone.0224059.s001.docx]

**Table S1 – Sociodemographic and “Willingness to accept” variables, questions and scales used in the questionnaires.**

| Variables | Questions | Scales |
| --- | --- | --- |
| Age | How old are you? | years |
| Gender | Gender | 1:male; 2:female |
| Education | What is your education level? | 1:incomplete elementary school;  2:complete elementary school;  3:incomplete high school;  4:complete high school;  5:incomplete bachelor degree;  6:complete bachelor degree;  7:incomplete postgraduate studies;  8:complete postgraduate studies |
| Income | What is your monthly income ? | 1: more than R$14.970,00; 2: R$4.990,00 – R$14.970,00; 3: R$2.994,00 – R$4.970,00; 4: R$998,00 - R$2.994,00; 5: R$998,00 |
| Contact with farm animals | Have you ever had contact with poultry ^a^ farms? | 0:no; 1:yes |
| Type of contact with farm animals | What type of contact did you have with poultry ^a^ farms? | 1: I lived in a rural propriety that produced broilers ^a^; 2: Someone in the family had or has a rural property that produces broiler ^a^; 3: I visited rural properties that produced broilers ^a^, but I never had direct contact with these animals; 4: I work or worked in poultry ^a^ supply chain; 5: other |
| Local of residence | Do you live in urban or rural area? | 1: urban; 2:rural; 3:both |
| Region | What region of the country do you live? | 0: developed (Southeast and South); 2: in development (Midwest; Northeast; North) |
| Willingness to accept | Would you be willing to accept the use of insects in poultry^a^ feed? | 0:no; 1:yes |

^a^ The words ‘poultry or broiler’ was replaced by the word ‘beef or cattle’ in the beef questionnaire, by the word ‘pig or pork’ in the pig questionnaire and by the word ‘fish’ in the fish questionnaire.
