# Supplementary material for: Understanding the factors influencing consumer willingness to accept the use of insects to feed poultry, cattle, pigs and fish in Brazil
Source: PLoS One. 2020 Apr 30;15(4):e0224059. doi: 10.1371/journal.pone.0224059 (PMC7192463; doi:10.1371/journal.pone.0224059)
Supplement: S2 Table — (DOCX) [file pone.0224059.s002.docx]

**Table S2 – Questions and scales used to measure attitude variables.**

| Variables | Questions | Scales |
| --- | --- | --- |
| Attitude 1 | What do you think about the idea of producing insects instead of grains for use in animal feed? | 1:negative; 5:positive |
| Attitude 2 | What do you think about the idea of producing insects instead of grains for use in animal feed? | 1:bad; 5:good |
| Attitude 3 | What do you think about the idea of producing insects instead of grains for use in animal feed? | 1:uneasy; 5:easy |
| Attitude 4 | What do you think about the idea of producing insects instead of grains for use in animal feed? | 1:not satisfied; 5:satisfied |
| Attitude 5 | What do you think about the idea of using insects as ingredient in animal feed? | 1:negative; 5:positive |
| Attitude 6 | What do you think about the idea of using insects as ingredient in animal feed? | 1:bad; 5:good |
| Attitude 7 | What do you think about the idea of using insects as ingredient in animal feed? | 1:uneasy; 5:easy |
| Attitude 8 | What do you think about the idea of using insects as ingredient in animal feed? | 1:not satisfied; 5:satisfied |
| Attitude 9 | What do you think about the idea of using insects in poultry ^a^ feed? | 1:negative; 5:positive |
| Attitude 10 | What do you think about the idea of using insects in poultry ^a^ feed? | 1:bad; 5:good |
| Attitude 11 | What do you think about the idea of using insects in poultry ^a^ feed? | 1:uneasy; 5:easy |
| Attitude 12 | What do you think about the idea of using insects in poultry ^a^ feed? | 1:not satisfied; 5:satisfied |

Adapted from Verbeke et al. (2015). ^a^ The word ‘poultry’ was replaced by the word ‘beef or cattle’ in the beef questionnaire, by the word ‘pig or pork’ in the pig questionnaire and by the word ‘fish’ in the fish questionnaire.
