# Supplementary material for: Understanding the factors influencing consumer willingness to accept the use of insects to feed poultry, cattle, pigs and fish in Brazil
Source: PLoS One. 2020 Apr 30;15(4):e0224059. doi: 10.1371/journal.pone.0224059 (PMC7192463; doi:10.1371/journal.pone.0224059)
Supplement: S3 Table — (DOCX) [file pone.0224059.s003.docx]

**Table S3 – Questions and scales used to measure the perceived benefits and perceived risks variables.**

| Variables | Questions | Scales |
| --- | --- | --- |
| Perception of benefits 1 | The use of insect in poultry ^a^ feed could allow organic waste to be better valorized. | 1:totally disagree; 2: disagree; 3:neither agree nor disagree, 4: agree, and 5: totally agree |
| Perception of benefits 2 | The use of insect in poultry ^a^ feed could allow sustainability to be improved. | 1:totally disagree; 2: disagree; 3:neither agree nor disagree, 4: agree, and 5: totally agree |
| Perception of benefits 3 | The use of insect in poultry ^a^ feed could allow the production of enough food to world population. | 1:totally disagree; 2: disagree; 3:neither agree nor disagree, 4: agree, and 5: totally agree |
| Perception of benefits 4 | The use of insects in poultry ^a^ feed may reduce the price of feed and animal production. | 1:totally disagree; 2: disagree; 3:neither agree nor disagree, 4: agree, and 5: totally agree |
| Perception of benefits 5 | The use of insects in poultry ^a^ feed can improve society's acceptance of poultry production. | 1:totally disagree; 2: disagree; 3:neither agree nor disagree, 4: agree, and 5: totally agree |
| Perception of risks 1 | The use of insects in poultry ^a^ feed may cause allergic reactions in humans. | 1:totally disagree; 2: disagree; 3:neither agree nor disagree, 4: agree, and 5: totally agree |
| Perception of risks 2 | The use of insects in poultry ^a^ feed may cause allergic reactions in animals. | 1:totally disagree; 2: disagree; 3:neither agree nor disagree, 4: agree, and 5: totally agree |
| Perception of risks 3 | The use of insects in poultry ^a^ feed can impact on biodiversity if the insects are accidentally released. | 1:totally disagree; 2: disagree; 3:neither agree nor disagree, 4: agree, and 5: totally agree |
| Perception of risks 4 | The use of insects in poultry ^a^ feed may introduce microbiological contamination in food supply chain. | 1:totally disagree; 2: disagree; 3:neither agree nor disagree, 4: agree, and 5: totally agree |
| Perception of risks 5 | The use of insects in poultry ^a^ feed can increase competitiveness with other agricultural activities. | 1:totally disagree; 2: disagree; 3:neither agree nor disagree, 4: agree, and 5: totally agree |
| Perception of risks 6 | The use of insects in poultry ^a^ feed may reduce the consumers acceptance of food resulting from animal production. | 1:totally disagree; 2: disagree; 3:neither agree nor disagree, 4: agree, and 5: totally agree |
| Perception of risks 7 | The use of insects in poultry ^a^ feed can introduce chemical residues into the food supply chain. | 1:totally disagree; 2: disagree; 3:neither agree nor disagree, 4: agree, and 5: totally agree |

Adapted from Verbeke et al. (2015). ^a^ The word ‘poultry’ was replaced by the word ‘beef or cattle’ in the beef questionnaire, by the word ‘pig or pork’ in the pig questionnaire and by the word ‘fish’ in the fish questionnaire.
