# Supplementary material for: Understanding the factors influencing consumer willingness to accept the use of insects to feed poultry, cattle, pigs and fish in Brazil
Source: PLoS One. 2020 Apr 30;15(4):e0224059. doi: 10.1371/journal.pone.0224059 (PMC7192463; doi:10.1371/journal.pone.0224059)
Supplement: S4 Table — (DOCX) [file pone.0224059.s004.docx]

**Table S4 – Questions and scales used to measure perceived challenges variables.**

| Variables | Questions | Scales |
| --- | --- | --- |
|  | To what extent are you concern about… |  |
| Challenge 1 | …consumer acceptance when insects are used in poultry ^a^ feed? | 1:not concern at all; 2: rather not concerned; 3:neither agree nor disagree, 4: rather concerned, and 5: very much concerned |
| Challenge 2 | …legislation when insects are used in poultry ^a^ feed? | 1:not concern at all; 2: rather not concerned; 3:neither agree nor disagree, 4: rather concerned, and 5: very much concerned |
| Challenge 3 | …the communication with consumers when insects are used in poultry ^a^ feed? | 1:not concern at all; 2: rather not concerned; 3:neither agree nor disagree, 4: rather concerned, and 5: very much concerned |
| Challenge 4 | …the communication with farmers when insects are used in poultry ^a^ feed? | 1:not concern at all; 2: rather not concerned; 3:neither agree nor disagree, 4: rather concerned, and 5: very much concerned |
| Challenge 5 | …sanitary policy and inspection when insects are used in poultry ^a^ feed? | 1:not concern at all; 2: rather not concerned; 3:neither agree nor disagree, 4: rather concerned, and 5: very much concerned |
| Challenge 6 | …food packaging when insects are used in poultry ^a^ feed? | 1:not concern at all; 2: rather not concerned; 3:neither agree nor disagree, 4: rather concerned, and 5: very much concerned |
| Challenge 7 | …ensuring enough insects to supply the demand when insects are used in poultry ^a^ feed? | 1:not concern at all; 2: rather not concerned; 3:neither agree nor disagree, 4: rather concerned, and 5: very much concerned |
| Challenge 8 | …how insects will be reared when they are used in poultry ^a^ feed? | 1:not concern at all; 2: rather not concerned; 3:neither agree nor disagree, 4: rather concerned, and 5: very much concerned |
| Challenge 9 | …how insects will be processed when they are used in poultry ^a^ feed? | 1:not concern at all; 2: rather not concerned; 3:neither agree nor disagree, 4: rather concerned, and 5: very much concerned |
| Challenge 10 | …feed quality when insects are used in poultry ^a^ feed? | 1:not concern at all; 2: rather not concerned; 3:neither agree nor disagree, 4: rather concerned, and 5: very much concerned |

Adapted from Verbeke et al. (2015). ^a^ The word ‘poultry’ was replaced by the word ‘beef or cattle’ in the beef questionnaire, by the word ‘pig or pork’ in the pig questionnaire and by the word ‘fish’ in the fish questionnaire.
