# Supplementary material for: Understanding the factors influencing consumer willingness to accept the use of insects to feed poultry, cattle, pigs and fish in Brazil
Source: PLoS One. 2020 Apr 30;15(4):e0224059. doi: 10.1371/journal.pone.0224059 (PMC7192463; doi:10.1371/journal.pone.0224059)
Supplement: S5 Table — (DOCX) [file pone.0224059.s005.docx]

**Table S5 – Descriptive statistics of attitude items used in the poultry, cattle, pig and fish questionnaires.**

| Items | Statements/Scales | Poultry  Mean (SD) | Cattle  Mean (SD) | Pig  Mean (SD) | Fish  Mean (SD) |
| --- | --- | --- | --- | --- | --- |
|  | What do you think of the idea about producing insects instead of grains for use in animal feed? |  |  |  |  |
| Att 1 | 1:negative; 5:positive | 2.72 (0.10) | 2.56 (0.10) | 2.70 (0.10) | 3 (0.11) |
| Att 2 | 1:bad; 5:good | 2.74 (0.11) | 2.50 (0.10) | 2.63 (0.11) | 3.16 (0.11) |
| Att 3 | 1:uneasy; 5:easy | 2.76 (0.12) | 2.50 (0.11) | 2.66 (0.11) | 2.86 (0.10) |
| Att 4 | 1:not satisfied; 5:satisfied | 2.68 (0.11) | 2.40 (0.10) | 2.52 (0.10) | 2.97 (0.10) |
|  | What do you think about the idea of using insects as ingredient in animal feed? |  |  |  |  |
| Att 5 | 1:negative; 5:positive | 2.88 (0.11) | 2.71 (0.11) | 2.69 (0.11) | 3.08 (0.11) |
| Att 6 | 1:bad; 5:good | 2.84 (0.11) | 2.58 (0.11) | 2.75 (0.11) | 3.17 (0.11) |
| Att 7 | 1:uneasy; 5:easy | 2.71 (0.10) | 2.62 (0.11) | 2.74 (0.11) | 3.11 (0.11) |
| Att 8 | 1:not satisfied; 5:satisfied | 2.82 (0.12) | 2.56 (0.10) | 2.63 (0.10) | 3.08 (0.11) |
|  | What do you think about the idea of using insects in poultry ^a^ feed? |  |  |  |  |
| Att 9 | 1:negative; 5:positive | 2.82 (0.12) | 2.62 (0.11) | 2.77 (0.11) | 3.44 (0.12) |
| Att 10 | 1:bad; 5:good | 2.90 (0.11) | 2.70 (0.12) | 2.95 (0.12) | 3.46 (0.12) |
| Att 11 | 1:uneasy; 5:easy | 2.9 (0.12) | 2.59 (0.11) | 2.66 (0.11) | 3.39 (0.11) |
| Att 12 | 1:not satisfied; 5:satisfied | 2.83 (0.12) | 2.53 (0.11) | 2.74 (0.11) | 3.26 (0.11) |

^a^ The words ‘poultry or broiler’ was replaced by the word ‘beef or cattle’ in the beef questionnaire, by the word ‘pig or pork’ in the pig questionnaire and by the word ‘fish’ in the fish questionnaire.
