# Supplementary material for: Understanding the factors influencing consumer willingness to accept the use of insects to feed poultry, cattle, pigs and fish in Brazil
Source: PLoS One. 2020 Apr 30;15(4):e0224059. doi: 10.1371/journal.pone.0224059 (PMC7192463; doi:10.1371/journal.pone.0224059)
Supplement: S6 Table — (DOCX) [file pone.0224059.s006.docx]

**Table S6 – Descriptive statistics of perception items used in the poultry, cattle, pig and fish questionnaires.**

| Items | Statements^a^ | Poultry  Mean (SD) | Cattle  Mean (SD) | Pig  Mean (SD) | Fish  Mean (SD) |
| --- | --- | --- | --- | --- | --- |
| Benefits | The use of insect in poultry^b^ feed… |  |  |  |  |
| PB1 | Could allow organic waste to be better valorized | 3.02 (0.11) | 2.64 (0.11) | 2.93 (0.11) | 3.35 (0.10) |
| PB2 | Could allow sustainability to be improved | 3.02 (0.11) | 2.84 (0.11) | 2.93 (0.11) | 3.46 (0.11) |
| PB3 | Could allow the production of enough food to world population | 2.86 (0.11) | 2.63 (0.11) | 2.79 (0.11) | 3.23 (0.10) |
| PB4 | May reduce the price of feed and animal production | 3.25 (0.11) | 3.07 (0.11) | 3.18 (0.11) | 3.42 (0.10) |
| PB5 | Can improve society's acceptance of poultry production | 2.54 (0.10) | 2.32 (0.10) | 2.50 (0.10) | 2.99 (0.10) |
| Risks | The use of insect in poultry^b^ feed… |  |  |  |  |
| PR1 | May cause allergic reactions in humans | 2.83 (0.12) | 3.02 (0.11) | 3.08 (0.11) | 2.68 (0.10) |
| PR2 | May cause allergic reactions in animals | 3.32 (0.11) | 3.42 (0.11) | 3.29 (0.11) | 3.42 (0.10) |
| PR3 | Can impact on biodiversity if the insects are accidentally released | 2.97 (0.11) | 3.18 (0.11) | 3.32 (0.10) | 3.09 (0.11) |
| PR4 | May introduce microbiological contamination in food supply chain | 2.94 (0.11) | 3.02 (0.11) | 3.16 (0.11) | 2.82 (0.11) |
| PR5 | Can increase competitiveness with other agricultural activities | 2.93 (0.10) | 2.83 (0.11) | 3.04 (0.11) | 3.08 (0.10) |
| PR6 | May reduce the consumers acceptance of food resulting from animal production | 3.14 (0.10) | 3.35 (0.11) | 3.35 (0.11) | 3.18 (0.10) |
| PR7 | Can introduce chemical residues into the food supply chain | 2.74 (0.11) | 3.11 (0.11) | 3.04 (0.11) | 2.83 (0.10) |

^a^ All the statements were measured using a Likert-type scale (1:strongly disagree; 2:disagree; 3:neutral; 4:agree; 5:strongly agree); ^b^ The words ‘poultry or broiler’ was replaced by the word ‘beef or cattle’ in the beef questionnaire, by the word ‘pig or pork’ in the pig questionnaire and by the word ‘fish’ in the fish questionnaire.
