# Supplementary material for: Understanding the factors influencing consumer willingness to accept the use of insects to feed poultry, cattle, pigs and fish in Brazil
Source: PLoS One. 2020 Apr 30;15(4):e0224059. doi: 10.1371/journal.pone.0224059 (PMC7192463; doi:10.1371/journal.pone.0224059)
Supplement: S7 Table — (DOCX) [file pone.0224059.s007.docx]

**Table S7 – Descriptive statistics of challenges items used in the poultry, cattle, pig and fish questionnaires.**

| Items | Statements^a^ | Poultry  Mean (SD) | Cattle  Mean (SD) | Pig  Mean (SD) | Fish  Mean (SD) |
| --- | --- | --- | --- | --- | --- |
| Challenges | To what extent are you concern about… |  |  |  |  |
| C1 | ...consumer acceptance when insects are used in poultry ^a^ feed? | 3.46 (0.10) | 3.46 (0.11) | 3.42 (0.10) | 3.05 (0.10) |
| C2 | …legislation when insects are used in poultry ^a^ feed? | 3.53 (0.10) | 3.58 (0.11) | 3.66 (0.10) | 3.22 (0.11) |
| C3 | …the communication with consumers when insects are used in poultry ^a^ feed? | 3.70 (0.10) | 3.76 (0.10) | 3.73 (0.10) | 3.42 (0.10) |
| C4 | …the communication with farmers when insects are used in poultry ^a^ feed? | 3.54 (0.10) | 3.76 (0.10) | 3.66 (0.10) | 3.28 (0.10) |
| C5 | …sanitary policy and inspection when insects are used in poultry ^a^ feed? | 4.16 (0.09) | 4.05 (0.10) | 4.10 (0.09) | 3.64 (0.10) |
| C6 | …food packaging when insects are used in poultry ^a^ feed? | 3.76 (0.10) | 3.78 (0.11) | 3.66 (0.10) | 3.42 (0.10) |
| C7 | …ensuring enough insects to supply the demand when insects are used in poultry ^a^ feed? | 3.56 (0.10) | 3.42 (0.11) | 3.51 (0.10) | 3.32 (0.11) |
| C8 | …how insects will be reared when they are used in poultry ^a^ feed? | 3.84 (0.10) | 3.99 (0.10) | 3.84 (0.10) | 3.90 (0.10) |
| C9 | …how insects will be processed when they are used in poultry ^a^ feed? | 3.82 (0.10) | 3.93 (0.10) | 3.78 (0.10) | 3.74 (0.10) |
| C10 | …feed quality when insects are used in poultry ^a^ feed? | 3.97 (0.09) | 3.96 (0.10) | 3.92 (0.09 | 3.70 (0.11) |

^a^ All the statements were measured using a Likert-type scale (1: not concern at all; 2: rather not concerned; 3:neither agree nor disagree, 4: rather concerned, and 5: very much concerned); ^b^ The words ‘poultry or broiler’ was replaced by the word ‘beef or cattle’ in the beef questionnaire, by the word ‘pig or pork’ in the pig questionnaire and by the word ‘fish’ in the fish questionnaire.
